# “They call me a glue kid, but I don’t like it”: Experiences of glue use among street-connected adolescents in Merauke Regency, Indonesia

**DOI:** 10.1371/journal.pone.0359626

**Published:** 2026-10-05

**Authors:** Faisal Adi Irawan, Ferry Efendi, Praba Diyan Rachmawati

**Affiliations:** 1 Magister of Nursing Student, Faculty of Nursing, Universitas Airlangga, Surabaya, Indonesia; 2 Faculty of Nursing, Universitas Airlangga, Surabaya, Indonesia; 3 Research Center in Advancing Community Healthcare (REACH), Universitas Airlangga, Surabaya, Indonesia; 4 School of Nursing and Midwifery, La Trobe University, Melbourne, Australia; 5 College of Nursing, Kaohsiung Medical University, Kaohsiung, Taiwan; Touro University California College of Pharmacy, UNITED STATES OF AMERICA

## Abstract

**Background:**

Glue-sniffing among street-connected children and adolescents is a neglected public health and social issue with acute toxicity, entrenched stigma, and long-term life-course harms. In Merauke (South Papua), everyday practices, peer dynamics, and social labeling remain under-described.

**Objective:**

To explore lived experiences of street-connected children and adolescents who use inhalants in Merauke, focusing on initiation, accessibility, peer processes, harms, return to glue use, and perceived stigma.

**Methods:**

We conducted an inductive descriptive qualitative study with children and adolescents aged 12–18 years who reported glue use in the prior three months. Participants were recruited via gatekeeper-facilitated snowball sampling until information power indicated sufficiency. Data comprised semi-structured in-depth interviews (audio-recorded) and field notes; brief member checking closed each session and after the analysis. Transcripts were analyzed using inductive content analysis in NVivo 15.

**Results:**

Twenty-two participants (19 male, 3 female) shared narratives; among the nine who recalled their age at initiation, first glue use occurred between 5 and 12 years. Participants reported inhaling commercially available adhesive products. Five categories emerged: (1) Early Exposure, Easy Access, and Peer Influence (Early Initiation, Easy Availability, and coercive invitations); (2) Everyday Glue Use and Its consequences (earmarking daily income for glue, polysubstance patterns, concealment, somatic/perceptual disturbances); (3) Social dynamics and peer support (in group solidarity, ambivalent community/enforcement responses, painful labeling); (4) Returning to the cycle (peer-triggered return, difficulty quitting, and strong attachment to glue); and (5) Hopes and multilevel strategies (age-gated sales and basic-needs support).

**Conclusions:**

Glue-sniffing in Merauke is sustained by permissive retail environments and peer-mediated cues set against structural deprivation. Effective prevention requires integrated policy, peer, service, and social-protection responses tailored to local contexts.

## Introduction

Glue-sniffing remains a serious yet under-recognized public health and social problem among street-connected children and adolescents in many low- and middle-income countries, including Thailand, Indonesia, Cambodia, Malaysia, Pakistan, Bangladesh, India, and the Philippines [1–3]. Among drug users identified in Indonesia’s 2023 national survey, 2.77% reported using substances within a combined category that included datura, LSD, psilocybin mushrooms, and inhaled intoxicants such as glue, gasoline, and markers [4]. Glue use among street-connected children and adolescents has also been documented in Merauke, with a significant proportion reportedly engaging in inhalant use [5]. Glue-sniffing is often viewed as a behavioral deviation, yet it reflects broader social and economic vulnerabilities that expose children and adolescents to multiple health risks, stigmatization, and marginalization [6].

Motivations for glue use are multifaceted and often shaped by the hardships of street life. Common reasons include curiosity about its intoxicating effects, temporary relief from hunger and fatigue, and a means of escaping stress or emotional distress [7–11]. The practice causes a wide range of health problems, such as neurological impairment, respiratory damage, and sudden sniffing death, all of which warrant urgent service attention [1,2,12]. On a social level, stigmatizing labels such as “glue kid” or “aibon kid” often lead to verbal abuse, rejection, and neglect, further excluding them from community support and formal services [13]. This reality contradicts the Sustainable Development Goal 3 mandate to promote health and well-being for all [14], as glue-sniffing exposes an already vulnerable group to preventable physical, psychosocial, and developmental harms while limiting their opportunity to achieve healthy development and well-being [15]. This is particularly relevant to the present study, as it highlights glue-sniffing not only as an individual behavioral issue, but also as a broader public health and social equity concern affecting marginalized adolescents.

Environmental and structural factors further sustain the practice. Glue products are cheap, legal, and easily available in local shops without age restrictions, allowing children and adolescents to obtain them freely. The low cost and unrestricted access make glue a common alternative for those unable to afford narcotics or alcohol [16–18]. Peer influence and social imitation play a central role in initiation and continuation, while the absence of parental supervision, educational opportunities, and safe spaces increases vulnerability. Street-connected children and adolescents, typically aged between 12 and 18 years, experience unstable living conditions and a constant need to adapt to social pressures within their peer groups [19,20].

Despite its persistence, research on glue-sniffing in Indonesia, especially in remote and border regions such as Merauke Regency in South Papua remains scarce. Existing studies predominantly document prevalence or medical effects, offering limited understanding of the lived experiences and social realities of children and adolescents involved in glue-sniffing. A deeper qualitative exploration is necessary to understand how these children and adolescents begin and sustain glue use, how they perceive its health and psychosocial impacts, and how they navigate stigma and social exclusion in daily life. Understanding these local experiences in Merauke is important because glue-sniffing among street-connected adolescents is shaped by social, cultural, and structural conditions that may differ across settings. Insights from this context can help inform more context-sensitive and culturally appropriate prevention and support strategies for marginalized adolescents in other Indonesian and Southeast Asian settings facing similar vulnerabilities. These interacting influences can be understood through Bronfenbrenner’s Ecological Systems Theory, which conceptualizes adolescent behavior as shaped by nested environmental systems ranging from immediate social relationships to broader structural and cultural contexts [21]. An ecological perspective is also widely used in adolescent substance-use research, where risk and protective factors are understood across multiple developmental contexts, including peers, family, school, and community environments [22].

Therefore, this study aims to explore the lived experiences of street-connected children and adolescents who use inhalants in Merauke, Indonesia. It focuses on initiation processes, patterns of use, perceived consequences, interactions with peers and community members, and expectations for prevention and support services.

## Materials and methods

### Study design

This study employed a descriptive qualitative design to explore and understand the lived experiences of street-connected children and adolescents engaged in inhalant (glue-sniffing) use in Merauke, Indonesia, consistent with qualitative description as an approach suited to capturing participants experiences in everyday terms [23]. The approach allowed an in-depth exploration of subjective meanings surrounding initiation, continuation, and adaptation to glue use within their everyday social environments. The study was guided by a naturalistic paradigm, emphasizing participants’ voices and contextual interpretation of their lived realities.

### Population, sample, and sampling

The unit of analysis was street-connected children and adolescents in Merauke who reported a history of inhalant use. Operationally, *street-connecteds* were defined as those who spent time in public spaces every day to earn money and socialize or play, despite maintaining varying ties to a household. Eligibility and contextual information were established with support from the gatekeeper and referrals during snowball recruitment. An *inhalant user* was defined as a child and adolescent who self-reported glue use within the past three months.

Inclusion criteria included: (1) aged 12–18 years; (2) currently using or having recently used glue; (3) able to communicate in Indonesian or a locally understood language; (4) willing to participate and schedule interviews; and (5) not under the influence of substances at the time of data collection. Exclusion criteria were physical or psychosocial conditions that could compromise safety or hinder meaningful participation.

Sampling followed a snowball strategy facilitated by local gatekeepers from the Department of Social Affairs. These gatekeepers were outreach workers or assistants recommended by the Department of Social Affairs who had routine interaction with, and close emotional ties to, street-connected children and adolescents. They identified the initial potential participants based on their field knowledge and daily interactions with these participants. Because snowball sampling was employed, the gatekeepers identified and recommended potential participants through their initial social networks. In this setting, street-connected children and adolescents tended to gather in groups or clusters with peers who shared similar glue-sniffing behaviors and regularly used inhalants together. The gatekeepers had established connections with these participants across the study locations, as they had known many of them since childhood, before the onset of glue-sniffing, and had remained familiar with them over time. The gatekeepers then introduced the study and facilitated the first contact between the researcher and the potential participants. Of the 23 eligible individuals approached, one declined to participate because they did not wish to be interviewed without a trusted companion when the gatekeeper was temporarily unavailable. This decision was respected, resulting in a final sample of 22 participants. No participant withdrew after enrollment. Gatekeepers initially identified the first participant, after which peer referrals were used to reach additional participants, with gatekeeper verification against inclusion criteria. The initial candidate was identified through communication with the gatekeepers, who were asked to locate one potential respondent meeting the inclusion criteria. The first respondent was then directly verified by the researcher and gatekeeper by asking about age, most recent glue use, willingness to participate in an interview, and whether the respondent had used glue shortly before the interview, as this could affect the quality of data collection. Communication ability was also assessed jointly by the researcher and gatekeeper to ensure that the respondent was able to participate meaningfully in the interview. This approach was selected because street-connected adolescents with glue-sniffing behavior represented a vulnerable and hard-to-reach population, for whom access through trusted intermediaries was considered more feasible and ethically appropriate than direct recruitment by unfamiliar researchers. However, this strategy may also have limited access to participants outside existing referral networks. Subsequent participants were recruited in line with the snowball sampling principle, whereby participants who had already been interviewed recommended the next potential participants. These candidates were then verified by both the gatekeeper and the researcher during the recruitment and interview process to ensure that all interviewed participants met the predetermined inclusion criteria.

Sample adequacy was primarily determined by data saturation. The first author and research team determined that no substantively new information emerged from the 18th interview onward. Four subsequent interviews confirmed saturation, resulting in a final sample of 22 participants. Access procedures adhered to typical gathering points and schedules, and interviews were conducted in neutral, private, and participants safe locations. Researchers prioritized non-judgmental, empathetic communication throughout.

### Data collection

Data were collected through semi-structured, in-depth interviews, which served as the primary source of information, supported by field notes documenting nonverbal cues, the setting, and researcher reflections. All interviews were conducted by the first author, who was male. The first interview served as a pilot to refine the guide and probing techniques; it was retained in the dataset after quality assessment. Interviews were conducted in Indonesian, although participants occasionally used local terms. No formal interpreter was involved; gatekeepers assisted in clarifying the meaning of unfamiliar local expressions when necessary.

The flexible topic guide explored: (1) initiation and patterns of use; (2) motives and reinforcing factors;(3) peer dynamics and access to substances;(4) strategies for concealing or normalizing use;(5) experiences of stigma and interactions with community or authorities;(6) quitting attempts, glue-sniffing behavior triggers, and coping efforts; and (7) expectations for prevention and support. Commercial adhesive names were retained to preserve local context. Their chemical compositions were not independently verified, and no identical toxicological profile was assumed. Gatekeepers were present and could hear the interviews and member-checking discussions. Participants agreed to their presence and were reminded that participation was voluntary, their responses would not affect access to services, and they could request a private interview. Interviews were conducted in a private setting, and gatekeepers were instructed not to direct or influence participants’ responses.

All interviews were audio-recorded (with permission) and varied in duration from 60 to 90 minutes, depending on the adequacy of data obtained. Each participant was interviewed once, and no repeat interviews were conducted. Member checking was conducted in two stages: during the interviews, participants confirmed the interviewer’s summaries of their responses; after the analysis, they reviewed the final categories and interpretations to assess whether these reflected their experiences. The gatekeeper accompanied participants during both stages. Full interview transcripts were not returned to participants for comment or correction. Follow-up contacts were conducted when clarification was needed. The interview process began after obtaining permission from the Merauke Regency Department of Social Affairs on September 10, 2025. Initial engagement with street-connected children and adolescents engaged in glue sniffing took place on September 12, 2025, during which the first interviews with participants 1–3 were conducted. The overall interview process continued for 18 days and concluded on September 30, 2025. Interviews were transcribed verbatim, and fieldnotes were integrated to contextualize narratives. During de-identification, each participant was assigned a code using the letter R (respondent) followed by the interview sequence number, from the first to the twenty-second participant interviewed. This coding was used to protect participant confidentiality. Quotations were also selected carefully to avoid revealing participant identity. All data were stored securely by the researcher in an online storage system, with access restricted to the author team only. In addition, only the interviewer and the research team were able to access these research data using a password controlled by the researcher.

### Reflexivity

The first author, who conducted all interviews, has a nursing background and a specific research interest in the experiences of street-connected children and adolescents with glue-sniffing behavior. This interest was informed in part by the researcher’s familiarity with a setting where glue-sniffing among children and adolescents is a visible social concern, which contributed to contextual sensitivity but also required ongoing critical reflection throughout the study. The researcher was aware of the potential power imbalance and the possibility of socially desirable responses, particularly because the participants were a vulnerable group with limited prior involvement in research. The presence of both the researcher and the gatekeepers may also have influenced how openly participants shared their experiences. To address these concerns, the researcher adopted a non-judgmental and empathetic approach, used reflexive journaling to document assumptions and field impressions, and engaged in peer debriefing to examine potential bias during data collection and analysis. In addition, efforts were made to ensure participants’ comfort before the interviews, including providing food so that interviews were not conducted while participants were hungry or physically uncomfortable. These steps were intended to support a more respectful and trustworthy interview process rather than to influence participants’ responses.

### Data analysis

An inductive content analysis approach [23] was used to derive categories, sub-categories, and overarching categories directly from the data. After transcription, the first author repeatedly read the transcripts for immersion and identified meaning units relevant to the research focus. Following initial coding by the first author, selected transcripts, codes, and preliminary categories were reviewed by other members of the research team. The code book was iteratively revised, and differences in interpretation and final categories were resolved through team discussion and consensus. Fieldnotes and data that differed from the general patterns were also examined to refine the analysis and represent variations in participants’ experiences. Open coding was performed line-by-line, followed by iterative comparison, merging, and refinement of codes. Related codes were grouped into sub-categories, abstracted into categories, and then synthesized into broader categories that captured the core patterns of participants’ experiences. Representative quotations were used to illustrate the categories. The analytic process was supported by NVivo 15 (**Lumivero**) for systematic data management. Selected quotations were translated into English by several members of the research team. The translations were compared with the original Indonesian transcripts to preserve the intended meaning, including that of local expressions. Categories were developed inductively from participants’ accounts without using Ecological Systems Theory as an a priori coding framework. After the inductive analysis was completed, the theory was applied only as an interpretive framework in the Discussion to situate the findings across relevant ecological levels.

### Trustworthiness

Credibility was enhanced through in-depth interviewing with iterative probing, triangulation of interviews and field notes, peer debriefing (critical-friend), and direct member checking during the interview process after the analysis, in which participants were given opportunities to clarify their statements and to confirm, clarify, or correct the researcher’s summary of the information they had shared. Transferability was addressed via thick description of the Merauke context, participant characteristics, data-collection settings, and direct quotations reflecting experience ranges. Dependability was supported by a documented audit trail (guide versions, analytic decision logs, codebook revisions, debriefing notes) and consistent procedures from transcription to categories development. Confirmability was strengthened through a reflexive journal and transparent analytic evidence (nodes/codes, participant memos) managed in NVivo 15, with findings consistently linked to data excerpts.

### Ethical considerations

Ethical approval was granted by the Health Research Ethics Committee, Faculty of Nursing, Universitas Airlangga, Surabaya (No. 3832-KEPK, approval date: 9 September 2025). Participants aged 12–17 years provided written informed assent. Written surrogate permission was also obtained from a gatekeeper who had a longstanding caregiving relationship with the participants and had cared for them since childhood. The gatekeeper was formally recommended by the local Social Affairs Office and explicitly approved by the Research Ethics Committee as a surrogate consent provider. Participation proceeded only when both the participant’s voluntary assent and the gatekeeper’s written permission had been obtained.

Four participants were aged 18 years. After receiving complete information about the study, these participants personally and voluntarily agreed to participate. In accordance with the ethics committee-approved procedure, written permission from the gatekeeper was additionally obtained as an ethical safeguard because of the participants’ vulnerable and street-connected circumstances. This additional permission did not replace the participants’ own voluntary decisions. Photographs of the interview setting, researcher, and participants and/or gatekeepers were taken solely as administrative evidence of the interviews, not to document consent. Participants and gatekeepers were photographed from behind to conceal their faces and identifying features. Confidentiality, safety, and voluntary participation were ensured throughout the study. Participants were free to withdraw at any time without consequence. Confidentiality would only have been limited when serious health or safety risks required protective action, including acute health concerns, ongoing violence or exploitation, credible threats of retaliation, or risks arising from participation. No such disclosure or condition occurred, and no health or social referrals were required.

## Results

### Characteristic of participants

A total of 22 street-connected children and adolescents participated in the study, comprising 19 males and 3 females. Based on grouped age categories, 14 participants were aged 12–15 years and 8 were aged 16–18 years at the time of interview. Age at first glue inhalation was categorized as <10 years old for 4 participants and ≥10 years old for 5 participants, while 13 participants did not recall or did not specify their age of first use (see Table 1). Contextual information indicated that participants had discontinued schooling at the elementary level and lived with parents, relatives, or siblings. They spent time on the streets daily to earn money and play, generally returning home at night, although some occasionally stayed outside until morning after glue inhalation or alcohol consumption. They were reportedly street-connected from childhood and had never accessed formal social or health services, although the duration and family-contact patterns were not systematically recorded. Participants reported inhaling adhesive products sold under the commercial names Castol and Fox, including occasional use of both products. These names reflect participants’ reports, and the products’ chemical compositions were not independently verified. Most participants were engaged in informal work, while a small number were unemployed. The forms of informal work reported included port labor or stevedoring, ship crew assistance, parking work, casual or odd-job work, areca-nut selling, and scrap-metal collection.

**Table 1:** Characteristics of street-connected children and adolescents (n = 22).

Respondent Code	Gender *	Age (years old)	Age at First Glue Inhalation (years old)	Reported Adhesive Product **	Occupation ***
R1	M	12–15	≥10	Castol	Informal work
R2	M	12–15	Not recalled	Castol	Informal work
R3	M	16–18	≥10	Castol	Informal work
R4	M	16–18	Not recalled	Castol	Informal work
R5	M	16–18	Not recalled	Castol	Informal work
R6	M	16–18	Not recalled	Castol	Informal work
R7	M	16–18	<10	Castol	Informal work
R8	M	16–18	<10	Castol	Informal work
R9	M	12–15	Not recalled	Castol	Informal work
R10	M	16–18	<10	Castol	Informal work
R11	M	12–15	Not recalled	Castol	Informal work
R12	F	12–15	≥10	Castol	Informal work
R13	F	12–15	Not recalled	Castol	Informal work
R14	F	12–15	Not recalled	Castol	Informal work
R15	M	12–15	Not recalled	Castol	Unemployed
R16	M	12–15	Not recalled	Fox	Informal work
R17	M	12–15	≥10	Castol	Informal work
R18	M	16–18	≥10	Castol and Fox	Informal work
R19	M	12–15	<10	Castol	Informal work
R20	M	12–15	Not recalled	Castol	Unemployed
R21	M	12–15	Not recalled	Castol	Informal work
R22	M	12–15	Not recalled	Castol	Informal work

*: M = Male F = Female; **: Adhesive names were participant-reported; chemical composition and toxicological equivalence were not independently verified; ***: Informal work included port or ship labor, parking attendance, casual or odd jobs, areca-nut vending, and scrap-metal collection.

Analysis of the interview data generated five main categories and several related sub-categories that captured the core patterns in participants’ experiences of glue-sniffing. These categories and sub-categories are summarized in Table 2 to provide an overview of the analytic structure before each category is described in detail below.

**Table 2:** Categories and sub-categories.

No	Categories	Sub-categories
1	Early exposure, easy access, and peer influence	Early initiation of glue sniffing
		Easy access to glue
		Peer invitations and coercion to try glue
2	Everyday glue use and its consequences	Working for glue
		Increasingly irrational modes of use
		Hiding to avoid stigma
		Physical and mental disturbances
		Feeling intoxicated and its side effects
3	Social dynamics and peer support	Preference for friendships and solidarity
		Community and authorities’ responses
		Intergroup conflict or violence related to glue
		Labeling as a disliked reality
4	Returning to the same cycle	Repeated invitations from friends
		Not knowing how to quit
		Intention outweighs action
		Strong attachment to glue and no desire to stop
5	Hopes and Multi-Level Strategies for Preventing Glue-Sniffing	Structural policies that favor adolescent’s needs
		Holistic and inclusive implementation

### Category 1: Early exposure, easy access, and peer influence

This category describes how participants’ glue-sniffing behavior was shaped by early exposure, easy access to glue, and direct peer influence. Participants reported beginning glue sniffing at a young age, obtaining glue easily from local sellers, and being invited or pressured by peers to try it.

1.1 Early initiation of glue sniffing

This sub-category highlights that exposure to glue-sniffing often began at a very early age, suggesting that inhalant use had already entered participants’ social environments during childhood. One participant recalled first using glue at the age of seven, indicating how early this exposure could occur.

*“Yes, bro. the first time was when I was seven years old.”* (R19)

Another participant likewise described glue use as something that had started when they were still very young, reinforcing the pattern of early initiation during childhood.

*“Back when I was little… 12 years old… from Asmat, I had been sniffing glue since I was small.”* (R3)

1.2 Easy access to glue

Participant reported purchasing glue from nearby sellers without being questioned, which they perceived as making access routine and uncomplicated. Participants described how glue could be purchased with little scrutiny, including by using ordinary excuses to make the transaction appear acceptable. One participant explained that they often deceived sellers by claiming the glue was intended for shoe repair, even though it was actually purchased for inhalation.

*“They asked, what are you buying glue for? Usually we sometimes trick the markets seller too, then say the glue is for shoe repair… we usually trick the ‘uncle’ at the kiosk, say it’s for shoe glue even though we use it to inhale.* (R11)

Another participant described how nearby sellers provided glue without asking any questions reinforcing the perception that glue was easy to obtain.

*“At the markets near here. Near the Hulux Terminal over there in that area… they don’t ask, they just sold the glue without question.”* (R8)

1.3 Peer invitations and coercion to try glue

This sub-category shows that initiation into glue-sniffing was not always driven by curiosity alone, but could also involve coercion, repeated pressure, and fear of social or physical consequences if refusing. One participant described being forced by peers to try glue and feeling unable to refuse because of the threat of violence.

*“The boys are the ones who usually invite… they force… he keeps asking me. I said, if I don’t want to, but they force… if we don’t sniff, they’ll beat us.”* (R14)

A similar pattern was described by another participant, who recalled being pressured by an older individual and eventually using glue despite initial refusal.

*“Because an older brother told us to sniff… he forced it, he said this stuff is good… he said, just sniff this glue, it’s good, then I said I didn’t want to but they kept forcing me until I got drunk on glue like that..”* (R19)

### Category 2: Everyday glue use and its consequences

This category describes participants’ day-to-day experiences of glue-sniffing as part of routine life, including how money from informal work was used not only for basic needs but also to buy glue, how patterns of use could involve combining glue with other substances, and how use was often carried out in hidden places to avoid being seen or caught. Participants also described physical complaints and illness after use, alongside experiences of intoxication marked by dizziness, watery eyes, daydreaming, laughter, and vivid perceptions.

2.1 Working for glue

Income-generating activities were used to meet daily needs and to purchase glue. In one account, the participant described earning money from guarding parking, with daily amounts varying, and explained how the money was shared with parents while a portion was kept and used to buy glue.

*“Money from guarding parking there… it can be IDR 50,000, it can even be up to IDR 100,000… give it to the parents then give ourselves a little leftover… the money to go buy glue.”* (R14)

In another account, the participant described earnings based on “trips,” estimating a daily total and describing how that income was used for food and also for purchasing glue.

*“If 1 trip is IDR 60,000. If on average in 1 day it can be IDR 180,000… use it to buy food… and use it to buy glue as well.”* (R6)

2.2 Increasingly irrational modes of use

This sub-category illustrates that glue use was not always an isolated practice, but could develop into increasingly layered and irrational patterns involving other substances. Participants described combining glue with “sopi” (local alcohol) and cigarettes in ways that intensified intoxication and reinforced repeated use. One participant referred to this combined pattern as a “two-lane model,” in which glue-sniffing and drinking occurred in sequence.

*“For example we are sniffing like that. After that it feels kind of not drunk enough, so immediately add ‘sopi’… so like a two-lane model. After drinking ‘sopi’, then sniff glue… drunk in two lanes like that.”* (R7)

Another participant further described “two lanes” as sniffing glue and drinking, and added that cigarettes were usually involved, noting that glue alone was described as “not enough,” and the pattern could worsen when “sopi” was added.

*“So if two lanes it means sniffing glue and drinking, right. Well, usually if drinking there must be sniffing glue with cigarettes too… If glue alone is not enough. So it can get worse if adding ‘sopi’.”* (R21)

2.3 Hiding to avoid stigma

Use took place in concealed locations to avoid attention, arrest, or family surveillance. One participant described that sniffing usually happened in a quiet place and emphasized the need to hide, noting the risk of being caught and chased.

*“Usually when sniffing it is in a quiet place… yes and must hide. So cannot sniff visibly rather than get caught. Could be chased too.”* (R15)

Another participant described using the forest rather than open roadside areas near shops, explaining that they hid because they were afraid their parents would see them, and stated they never used in open places.

*“In the forest… not on the roads by the shop fronts like that. We usually hide, because we are afraid parents will see… never in such open places.”* (R18)

2.4 Physical and mental disturbances

Participants reported health complaints and deteriorating physical condition after use. One participant described experiencing heart illness and reported what the doctor said during an examination, including difficulty breathing.

*“I once because of heart illness, bro. So when the doctor examined, it was said the heart was dirty, black like that… breath gasping like that.”* (R15)

Another participant described becoming ill after being intoxicated from glue and sleeping, then waking up with body aches and chest pain, and also described having lung illness for two months, reporting that the doctor linked the illness to glue-sniffing.

*“There is illness, usually after getting drunk on glue then sleep… later when waking the body aches… chest hurts. I once had lung illness for 2 months before I recovered… the doctor said this illness was because of sniffing this glue.”* (R19)

2.5 Feeling intoxicated and its side effects

This sub-category illustrates how intoxication was subjectively experienced through dizziness, altered perception, and hallucination-like sensations. One participant described the bodily and perceptual effects of glue use as involving watery eyes, dizziness, daydreaming, and vivid imagination.

*“Head dizzy-dizzy, eyes watery, then daydreaming-daydreaming… laughing then imagining cars.”* (R10)

Another participant described similarly altered experiences, including sensations of flying, seeing unusual images, and laughing alone, reflecting the hallucinatory quality of intoxication.

*“Daydreaming-daydreaming… like we live above the earth, like we are flying … looking at something like that… like an angel, laughing to oneself.”* (R8)

### Category 3: Social dynamics and peer support

This category describes how participants’ social relationships and daily interactions were shaped by peer groups, community reactions, and responses from authorities. Across the sub-categories, participants described feeling more comfortable spending time with fellow glue users, sharing glue when it was scarce, experiencing community actions such as being given food instead of money, encountering chases, arrests, and physical punishment by authorities, facing conflict with other groups after use, and reacting emotionally to being labeled as “glue kid” or “aibon kid.”

3.1 Preference for friendships and solidarity

Friendships tended to form among fellow users because it felt more comfortable to interact and share experiences. One participant described feeling happier when spending time with “glue kids,” and described the interaction and conversation as pleasant when being with that group.

*“Like to hang out with the glue kids… more happy if hanging out just with them. Like talking that is pleasant like that.”* (R16)

In another account, a participant described that when glue was not available, they could ask a friend for a small amount to inhale, and described that this made it easier to talk and share with each other.

*“Yes like if for example there is no glue then can say, bro ask to inhale a little please… so it’s easier to talk for sharing with each other.”* (R18)

3.2 Community and authorities’ responses

This sub-category illustrates that participants encountered mixed responses from their surrounding environment, ranging from indirect forms of community concern to punitive actions by authorities. One participant described how community members sometimes avoided giving money and instead offered food, reflecting an ambivalent response that acknowledged need while attempting to limit substance use.

*“Different, they usually don’t give us money… then we only get given food.”* (R20)

In contrast, another participant described more coercive responses from authorities, including being chased, arrested, and physically punished, showing how institutional responses could intensify fear and marginalization.

*“Usually we are chased… arrested by police, soldiers too… police once… got hit-hit… beaten too… got told to squat for a very long time.”* (R15)

3.3 Intergroup conflict or violence related to glue

This sub-category shows that glue use could heighten vulnerability to conflict and aggression, particularly in peer-group interactions and intergroup confrontations. One participant described how a seemingly minor altercation escalated into a broader conflict involving another group after sniffing glue.

*“Got hit by a friend… once made a mess… war with a different complex… after sniffing glue… threw them with bricks.”* (R11)

Another participant described how violence could quickly escalate when intoxicated peers responded to trouble by returning with weapons to defend their group.

*“If when with friends they are drunk, then for example there is someone who makes trouble, we immediately go back to take a machete and help them again.”* (R22)

3.4 Labeling as a disliked reality

Labels like “glue kid/ aibon kid” evoked strong emotions, such as annoyance, anger, and sadness, and led to either confrontation or withdrawal. One participant described being called a glue kid, stated they did not like it, and described feeling annoyed and angry, including describing an urge to harm someone with a stone.

*“They call me a glue kid but I don’t like it… annoyed like that, angry… like wanting to kill like that… smash their head with a stone.”* (R9)

Another participant described being called an aibon kid, described feeling not good, heartache, and sadness, and then described leaving and walking away afterward.

*“Once, got called an aibon kid… feels kind of not good like that, like heartache like that, sad like that. After that I just went out walking.”* (R20)

### Category 4: Returning to the same cycle

This category describes how participants reported returning to glue use after attempts or intentions to stop. Across the sub-categories, participants described re-starting use after meeting friends who invited them again, expressing that they wanted to stop but did not know how, describing short pauses that did not last and were followed by using again the next day or when returning to the same place with money and glue available, and describing situations in which they did not want to stop because glue was already “too excessive” or “entered into the body,” including accounts of resisting parents’ anger or scolding.

4.1 Repeated invitations from friends

Attempts to quit often collapsed when re-encountering the old peer circle; sudden, everyday invitations triggered “starting again” despite prior abstinence or intentions to stop. One participant described that after returning to Merauke and having already stopped, friends asked them to buy glue, and they bought glue again.

*“When i returned to Merauke, i had already stopped, but friends asked me to buy glue, so we bought glue again.”* (R4)

Another participant described having the intention to stop the previous day, but described seeing friends in front of them, feeling influence, and ending up sniffing glue.

*“Yesterday actually there was an intention to stop sniffing glue. But seeing the friends in front of me there was influence there so ended up sniffing glue.”* (R7)

4.2 Not knowing how to quit

This sub-category highlights that the desire to stop using glue was often not accompanied by practical knowledge or strategies for quitting. One participant explicitly expressed a wish to stop while also admitting uncertainty about how to do so.

*“Yes, want to stop but still sniff, we do not know how.”* (R12)

Another participant similarly conveyed the absence of a clear way to quit, suggesting that repeated use continued not only because of habit and access, but also because stopping was not understood as a manageable process.

*“Cannot, bro. Do not know also how to stop.”* (R20)

4.3 Intention outweighs action

Brief “rests” were quickly broken by sleepiness, habit, available money, or place-based cues; intention exceeded action, so use recurred. One participant described that when they wanted to stop, they usually only rested, and at most stopped until night when they felt sleepy, but then sniffed again the next day.

*“If i want to stop, usually rest… at most until the night only if already sleepy put it away… yes tomorrow i sniff again.”* (R11)

Another participant described how available money, familiar places, and repeated return to the same environment made intentions to stop difficult to translate into action.

*“If on land continuously then we sniff continuously… but that’s if there is money, only then we can buy… because already stepped into the place again. This is the place for sniffing-sniffing, the stuff is already here.”* (R18)

4.4 Strong attachment to glue and no desire to stop

Some stated they did not want to stop because the attachment felt too strong; parental scolding or religious oaths were ineffective and could provoke resistance. One participant described not wanting to stop because it was already too excessive and hard to stop, and stated that being scolded by parents or oaths in church did not change the behavior.

*“Do not want to stop… because it’s already too excessive, right. So it’s hard to make it stop. Even if get scolded by parents or oaths in church it’s still the same still sniff that stuff.”* (R15)

Another participant described that there was no desire to stop because the glue had already entered the body and they could not stop, and also described that if parents were angry, they could fight back.

*“There isn’t either… because the glue has already entered into the body, so cannot stop. If parents can be angry, we can fight back.”* (R19)

### Category 5: Hopes and multi-level strategies for preventing glue-sniffing

This category describes participants’ expectations and suggested approaches for prevention. Across the sub-categories, participants described hopes that shops would not sell glue carelessly to children and adolescents, including statements about telling sellers not to sell and references to shops that are prohibited from selling glue. Participants also described prevention in terms of assistance and support such as school or dormitory options, as well as provision of basic needs including money, clothes, rice, and food, so that children and adolescents would not continue sniffing glue.

5.1 Structural policies that favor **adolescent**’**s** needs

It is expected that there will be clear and consistent regulation regarding distribution and sales of glue, especially preventing sales to children and adolescents. One participant described that shops should not sell carelessly because glue damages the lungs.

*“So that they don’t sell… because it damages the lungs, so that shops don’t sell carelessly.”* (R20)

Another participant described that sellers must be told not to casually sell glue to children, and stated that this had been the case for a long time, including mentioning some shops that are prohibited from selling glue.

*“Indeed they must be told not to casually sell glue to children. It’s been like that for a long time too, some shops that are prohibited from selling glue.”* (R18)

5.2 Holistic and inclusive implementation

Participants expressed the view that prevention should address education and basic needs simultaneously, including school or dormitory support, social assistance, food, and clothing. One participant described asking for help so that they do not inhale glue again, including giving them school or something like that, or providing a dormitory.

*“Maybe give departure or something… what’s important we can say to them… sir please can help them so that they don’t inhale glue again. Give them school or something like that, or please give them to stay in a dormitory.”* (R18)

Another participant emphasized the importance of meeting basic needs, suggesting that practical support such as money, clothing, and food was part of preventing continued glue use.

*“Like give money or give clothes… give rice, give food noodles like that. So that they, yes so that they don’t sniff-sniff continuously”* (R15)

## Discussion

This study provides a context-specific understanding of glue-sniffing behavior among street-connected children and adolescents in Merauke. Across the categories, three cross-cutting findings were apparent: early initiation, peer influence, and participants’ perceptions of easy access to glue interacted to embed its use in everyday social life; reported health and psychosocial consequences were compounded by stigma and limited support; and attempts to stop were difficult to sustain when participants returned to the same social and environmental contexts. These patterns suggest that glue-sniffing cannot be understood solely as an individual behavior but should be interpreted within the interacting interpersonal [12], community [24], and structural environments described by participants. This interpretation is consistent with Bronfenbrenner’s ecological systems theory, which conceptualizes development and behavior as emerging through interactions across nested environmental systems [21], and with research showing that youth substance use is shaped by risk and protective factors across peer, family, school, and leisure domains [22]. By demonstrating how these influences intersected in the everyday experiences of street-connected children and adolescents in Merauke, this study adds context-specific qualitative evidence to an inhalant literature in which the social and cultural dimensions of use in low- and middle-income countries remain under explored [25].

Early initiation appeared to emerge at the intersection of peer relationships and participants’ perceived access to commercial adhesive products. Among the nine participants who recalled their age at first use, initiation occurred between 5 and 12 years and was commonly described in connection with invitations or coercion from peers. Participants also reported purchasing glue from local shops with few questions, sometimes by presenting it as an adhesive for shoe repair. Within an ecological interpretation, peer invitations and direct encounters with sellers represented immediate social interactions, while the broader retail conditions perceived by participants formed part of the surrounding environment that may have reduced practical barriers to continued use. This combination may have enabled peer networks to lower the social threshold for experimentation while perceived ease of purchase lowered the practical threshold. This interpretation is consistent with evidence identifying availability, affordability, and peer influence as contributors to glue sniffing [18,26], systematic evidence that peer networks shape the initiation and continuation of adolescent substance use [7], and findings linking peer influence with adolescent risk-taking behavior [27,28]. Similar accounts of environmental influence and easy access have been reported among adolescents who inhaled glue in Manado [24]. However, because retailers were not interviewed, these findings should be understood as participants’ perceptions and reported purchasing experiences rather than independent verification of sales practices or regulatory enforcement.

Beyond initiation and access, participants’ accounts indicated that glue-sniffing had become interwoven with their daily economic and social routines. Some participants described dividing earnings from informal work between food, family contributions, and glue, while others reported using glue sequentially or together with cigarettes and locally produced alcohol. These accounts position glue use not as a discrete episode but as part of a broader pattern in which livelihood, substance use, and peer interaction overlapped. Acharya et al. [2] similarly found that street-connected children obtained money through street-based activities and identified belonging to a peer group as one reason for glue sniffing, while broader research indicates that polysubstance use is heterogeneous and may emerge during adolescence [5,13,29,30]. Within this immediate social environment, peer relationships played an ambivalent role: participants described friendship, comfort, conversation, and sharing glue when money or supplies were scarce, yet these same practices may have reduced material barriers to use and reinforced its normalization within the group. This interpretation is consistent with evidence that adolescent substance use is shaped through both peer selection and peer socialization processes [7,31,32]. It also supports calls to understand inhalant use among street-connected young people within the social and exploitative environments in which it occurs, rather than as an isolated individual deficit [11].

Yet the same everyday routines and peer relationships that provided companionship and practical support were also accompanied by physical, perceptual, and social consequences. Participants reported chest pain, breathing difficulties, body aches, dizziness, watery eyes, and hallucination-like experiences; however, these symptoms were self-reported and were not clinically assessed in this study. Comparable physical and mental manifestations have been described in Indonesian inhalant-related case literature [33,34], while serious hepatic and renal complications following glue inhalation have also been documented in a clinical case report [1]. These broader inhalant-related risks should not be interpreted as product-specific effects of Castol or Fox because these commercial names were reported by participants and the products’ chemical compositions were not independently verified. Socially, stigmatizing labels such as “glue kid” or “aibon kid” elicited anger, sadness, or withdrawal, while fear of family discovery, community judgment, or being pursued by authorities contributed to concealment. Participants also described some encounters with police or soldiers as frightening or punitive. These experiences are consistent with calls to understand inhalant use among street-involved young people within marginalizing environments characterized by limited supportive structures [11,31]. Nevertheless, the reported institutional encounters represent participants’ perspectives and were not corroborated with authorities; they therefore suggest possible barriers to trust and formal support rather than establishing institutional practices or effects.

Against this backdrop, participants’ efforts to stop revealed a further tension between their individual intentions and the social conditions surrounding glue use. Although some participants expressed a desire to stop, they described resuming use after meeting friends who used glue, receiving renewed invitations, or returning to familiar places where money and glue were available; several also stated that they did not know how to stop. From an ecological perspective, this pattern can be interpreted as a chronosystem process in which repeated exposure over time to the same microsystem, particularly peer groups and familiar gathering places, constrained participants’ efforts to translate their intentions into sustained action. Broader evidence similarly indicates that adolescent substance use is shaped through peer selection and socialization processes [7,31,35], while peer presence can influence adolescent risk-taking through cognitive-control processes [36]. In Manado, adolescents described concerns about health consequences, moral considerations, and fear of punishment as reasons for stopping glue use, suggesting that motivation to stop may coexist with social and environmental pressures that make cessation difficult to sustain [24]. Clinical literature also characterizes inhalant use as an often-overlooked problem that may require systematic assessment and multi component support [37,38]. Taken together, participants’ repeated return to glue use should not be viewed solely as an individual failure, but as a participant-reported behavioral pattern shaped by persistent peer exposure, reported limitations in cessation knowledge and support, and largely unchanged social and environmental contexts.

The difficulty of sustaining these individual efforts also helps explain why participants framed prevention in terms of changes to their surrounding conditions, rather than simply asking young people to stop using glue. They directly called for shops to avoid selling glue to children and adolescents and identified access to schooling, dormitory accommodation, food, clothing, and other basic assistance as forms of support that might help them stop. These priorities suggest that, from participants’ perspectives, prevention needed to address both access to glue and the educational, housing, and material conditions surrounding street-connected life. Their concerns about access are consistent with regional evidence identifying availability and affordability as factors contributing to glue sniffing and highlighting the associated challenges for health and social services [18]. Building on these participant-derived priorities, the researchers propose that retailer education and monitoring, youth-friendly health and social services, peer and family engagement, and coordination among health, educational, and social sectors could be considered as components of a locally adapted multilevel response. Community-engaged youth behavioral health initiatives emphasize that strong relationships, community partnerships, trust, and adaptation to local contexts and resources are important implementation conditions [39]. Research on drug-free community coalitions further indicates that prevention capacity and resources may be unequally distributed, underscoring the need to avoid assuming that strategies developed in better-resourced settings can be transferred directly to Merauke [40]. These broader responses were not directly proposed in full by participants but were derived by the researchers from participants’ stated priorities and the wider literature. Their feasibility, acceptability, and effectiveness were not assessed in this study and therefore require consultation with relevant stakeholders and formal evaluation.

The Merauke findings can be situated within a broader regional literature showing that glue-sniffing among street-connected children and adolescents is embedded in interacting social and structural conditions. Evidence from the SAARC region has identified availability, affordability, peer influence, and socioeconomic vulnerability as contributing factors [18], while research in Nepal has documented early initiation, participation in street-based peer groups, and associated health and social consequences [2]. Within Indonesia, adolescents in Manado similarly described environmental influence and easy access as factors shaping glue use, alongside health concerns, moral considerations, and fear of punishment as reasons for attempting to stop [24]. Research from India has further emphasized the need to interpret inhalant use within the marginalization and limited protective structures experienced by street-involved young people [11]. Rather than identifying factors that are necessarily unique to Merauke, the contribution of the present study lies in demonstrating how early initiation, peer relationships, participants’ perceived access to glue, stigma, material insecurity, and difficulty sustaining cessation intersected in the everyday accounts of street-connected children and adolescents in an under-researched eastern Indonesian setting. Because these findings were derived from participants recruited through gatekeeper-supported networks within one regency, they should not be generalized to all street-connected children and adolescents in Indonesia or other settings. Future research should incorporate the perspectives of families, retailers, health and social service providers, law-enforcement representatives, and policymakers, and should evaluate the feasibility, acceptability, and effectiveness of the responses proposed from the present findings.

### Relevance for clinical practice

This study provides essential insights for community nursing and pediatric mental health practice. Early exposure, easy access, peer coercion, stigma, and relapse patterns highlight the need for nurses to conduct early detection of inhalant use through observable physical and behavioral signs. Clinicians should apply non-stigmatizing, trauma-informed communication to support children who often experience labeling and social rejection.

For practice, collaboration with families, schools, and community workers is crucial to strengthen protective environments and reduce exposure risks. The findings also point to policy needs, particularly regulating glue sales to minors and providing education for retailers and community members. These measures can reinforce clinical efforts and improve safety and support for vulnerable street-connected children.

## Limitations

This study relied on snowball sampling facilitated by gatekeepers, which may have limited the diversity of participants and excluded street-connected children and adolescents outside established networks. In particular, participants who were more isolated, less connected to existing social networks, or not previously known to gatekeepers may have been underrepresented. As a result, the findings may not fully reflect the range of experiences among all street-connected adolescents with glue-sniffing behavior in Merauke, particularly those who remained outside established referral pathways. While this approach ensured trust and ethical access to a vulnerable population, it may have constrained the range of perspectives captured. In this study, street-connected adolescents with glue-sniffing behavior were a highly vulnerable and hard-to-reach population, and approaching them through someone they had not known or trusted over time would have been difficult. The involvement of gatekeepers therefore played an important role in facilitating initial contact, supporting a sensitive approach, and helping participants feel more comfortable. Gatekeepers also helped explain the purpose of the study and confirm whether the street-connected children and adolescents were willing to participate in the research or not. Gatekeepers also helped verify that participants met the intended inclusion criteria, thereby strengthening the appropriateness of recruitment for this study. For these reasons, the use of gatekeeper-facilitated sampling was considered the most feasible and ethically appropriate strategy for engaging participants who tended to be closed, difficult to approach, and in need of deeper rapport to generate open, meaningful, and varied accounts. Nevertheless, the method was appropriate for reaching hidden groups and yielded rich, contextually grounded insights. Detailed participant-level data on the duration of street connection, family contact, and overnight street stays were not systematically collected.

## Conclusion

This study demonstrates that glue-sniffing among street-connected children and adolescents in Merauke emerges from early initiation, participants’ perceptions of easy access to glue, peer reinforcement, and social neglect, with participants reporting health and psychosocial consequences as well as repeated returns to glue use after attempts to stop. The findings highlight that this phenomenon is not an isolated behavioral issue but a manifestation of structural vulnerability sustained by poverty, participants’ perceptions of limited restrictions on glue sales, and stigma. Effective prevention therefore requires multi-level strategies: enforcing restrictions on sales to minors through retailer monitoring, strengthening school–family–community partnerships to build coping and refusal skills, expanding youth-friendly and peer-led services, and ensuring access to education, safe housing, and basic needs. Cross-sector collaboration between health, social, and educational systems is essential to interrupt the cycle of repeated glue use and social exclusion, while positioning street-connected children and adolescents as active participants in designing interventions that restore dignity, protection, and long-term well-being. These broader responses represent implications derived from the participants’ accounts and require consultation with relevant stakeholders and further evaluation, as their effectiveness was not assessed in this study.
